# Supplementary material for: A Community Audit of 300 “Drop-Out” Instances in Children Undergoing Ponseti Clubfoot Care in Bangladesh—What Do the Parents Say?
Source: Int J Environ Res Public Health. 2021 Jan 23;18(3):993. doi: 10.3390/ijerph18030993 (PMC7908544; doi:10.3390/ijerph18030993)
Supplement: Supplementary file 1 [file ijerph-18-00993-s001.pdf]

Non clinical Questionnaire for assessment of Dropout cases during Home visit

We are very interested to know the parents comments about reasons for discontinuing their child's clubfoot treatment cycle. The questions below are based upon ways in which many parent didn't visit the clinic for treating their child's clubfoot usually after initial correction phase. Many of which may have develop full or partial relapse deformity.

- Patient's name: Age of started treatment: ID no:
- Father's Name: Contact no 1:
- Contact 2:
- Address: Full address including landmark
- Parent's occupation:
- How many members in your family?
- House made of: tin / wood / mud / brick / other……
- Do you live in single or combined family?
- How many members earning in your family?
- How old is your child now?
- How is your child feet now?
- When you stopped visiting for treatment of your child?
  - Casting phase
  - After tenotomy
  - After first brace fitting
  - Follow up phase
- Why you stopped continuing treatment of your child?
  - Due to poor financial condition
  - Treatment seems not effective
  - Long term treatment
  - Bad attitude of staffs during taking treatment
  - Difficult to visit long distance/ Transport difficulties
  - Not understanding importance brace use
  - Family problem
  - Forget appointment date
  - Family and community not well educated about clubfoot & long term treatment
  - Other………………………………

- Did you find any parts of the treatment difficult? Casts / tenotomy / tenotomy casts / brace / other…………..

- Does your child can bear weight on single foot/sit in Squat position symmetrically/ or you notice weakness in any side of legs and foot/ has your child had pain in their foot or ankle?

- What is the present condition of your child?(parents comments)
  - Very Good
  - Good
  - Average
  - Satisfactory
  - Not satisfactory

- If the condition is not satisfactory, Do you want to continue treatment again? Yes / No

- Do you need any kind of support? Yes/ No

- If yes, what kind of support you need?

- Is there anything else you want to share with me that we have not already discussed?

- Patient's present picture?

Thank you very much for your cooperation and the results of this interview will help us to manage other parents and children in our clubfoot clinics efficiently.

Signature/Name Parent ____
